# Statistical challenges in the development and evaluation of marker-based clinical tests

**DOI:** 10.1186/1741-7015-10-52

**Published:** 2012-05-29

**Authors:** Lisa M McShane

**Affiliations:** 1 Biometric Research Branch and Cancer Diagnosis Program, Division of Cancer Treatment and Diagnosis, National Cancer Institute, 6130 Executive Boulevard, EPN 8126, Bethesda, MD 20892-7434, USA

**Keywords:** Marker, biomarker, biostatistics, prognostic, predictive, treatment effect modifier, clinical test, translational research

## Abstract

Exciting new technologies for assessing markers in human specimens are now available to evaluate unprecedented types and numbers of variations in DNA, RNA, proteins, or biological structures such as chromosomes. These markers, whether viewed individually, or collectively as a 'signature', have the potential to be useful for disease risk assessment, screening, early detection, prognosis, therapy selection, and monitoring for therapy effectiveness or disease recurrence. Successful translation from basic research findings to clinically useful test requires basic, translational, and regulatory sciences and a collaborative effort among individuals with varied types of expertise including laboratory scientists, technology developers, clinicians, statisticians, and bioinformaticians. The focus of this commentary is the many statistical challenges in translational marker research, specifically in the development and validation of marker-based tests that have clinical utility for therapeutic decision-making.

## Introduction

It is increasingly recognized that many therapies will benefit only a subgroup of patients. Indeed, many new therapies are being engineered to target specific biological features of disease which, in turn, identify particular patient subgroups most likely to benefit from the therapy. In oncology, for example, the genetic makeup of a tumor might determine the biological pathways upon which it is dependent for survival and growth, and targeting components of that pathway with a new therapy might be the key to eradicating the tumor or arresting its growth [1]. Tumors not dependent on that pathway might be unaffected by the new therapy. Variations in germline DNA resulting in deficiency of drug metabolizing enzymes might dramatically influence a patient's risk of serious toxicities from certain drugs [2]. Therefore, clinical tests based on biological markers can be useful for making therapeutic decisions for patients newly diagnosed with a disease or for informing clinical management decisions throughout course of treatment.

The main types of marker-based tests for guiding therapy decisions at time of initial diagnosis are predictive tests, and to a lesser extent, prognostic tests [3]. In oncology terms, predictive markers are those associated with response (benefit) or lack of response to a particular therapy relative to other available therapy. In other medical specialties, terms such as treatment effect modifier, treatment-guiding marker, or treatment-selection marker are often used.

For some diseases there can be wide variation in the natural course of the disease process. Some patients may have very indolent disease with symptoms so mild that no, or minimal, therapy is recommended, whereas other patients might have a very aggressive form of the disease requiring intensive treatment. In these cases, prognostic markers may be useful in making these distinctions. These predict natural history of disease in the absence of further therapy. Sometimes this definition of prognostic marker is extended to natural history in the context of standard therapy that all patients are likely to receive. Marker-based tests that provide clinically important prognostic or predictive information are rapidly becoming integral to development and optimal utilization of therapeutics.

The need for rigorous statistical design and analysis methods has long been recognized in clinical trials for new therapeutics, but the same rigor often does not exist in the development and evaluation of marker-based tests. In oncology, there has been much discussion of the disappointing rate of progress in moving marker-based tests into clinical practice. The description provided in the American Society of Clinical Oncology 2007 Update of Recommendations for the Use of Tumor Markers in Breast Cancer typifies the situation: '... primary literature is characterized by studies that included small patient numbers, that are retrospective, and that commonly perform multiple analyses until one reveals a statistically significant result' [4]. This characterization is further supported by an oncology literature review conducted by Kyzas *et al. *[5] which found that 'almost all articles on cancer prognostic markers report statistically significant results'.

Few tumor prognostic markers ever make it into routine clinical practice to have an impact on patient care or outcomes. Although these examples come from oncology, the issues are widely applicable to marker research in other medical subspecialties. This commentary will explain how deficiencies in study design and statistical analysis methods have been large contributors to the modest rate of progress in translational marker research.

## Discussion

Marker studies should ideally be designed and conducted with a specific clinical question in mind just like therapeutic trials, but unfortunately, rigorously conducted marker studies seem to be the exception rather than the rule [6]. Many marker studies are conducted with a lack of attention to design and in the absence of a clinically meaningful marker question, that is, for what clinical use the marker is being considered or proposed [7]. Frequently marker studies are conducted retrospectively on 'convenience' specimen sets, which are specimen sets assembled based on availability, and may represent patients with highly diverse pathologic and clinical characteristics. The specimens may have been collected under unknown conditions, and the quality and completeness of associated clinical and pathological data may be unreliable. All of this heterogeneity makes it difficult to identify a coherent clinical setting in which the marker might be useful, even if the study is able to identify statistically significant associations between the marker and patient characteristics or outcomes.

### Ideal execution of retrospective prognostic marker studies

Specimens collected in the context of clinical trials, prospective cohort studies, or well-monitored prospective registries and which are stored for future use are the optimal source of specimens for retrospective prognostic studies [8]. Potential prognostic markers can be evaluated appropriately using specimens collected from patients enrolled on a placebo arm of a trial or an arm representing standard of care for the disease setting under study. Prognostic studies can also be conducted using specimens collected as part of prospective disease registries or from subjects in epidemiological study cohorts who were observed to develop disease if the clinical follow-up of the subjects who developed disease is sufficiently standardized and complete. Specimens collected from these sources are most likely to have been collected in a standardized fashion, the associated pathologic and clinical data tend to be most reliable, and therefore, the study is more likely to produce interpretable and convincing findings than studies conducted on convenience sets of specimens.

### Ideal execution of retrospective predictive marker studies

Predictive marker studies are most reliable when conducted using specimens that had been collected as part of a prospective clinical trial that randomized patients between a standard of care treatment and a new therapy for which the marker is being assessed for its predictive utility. Such specimen collections can be used to provide a high level of evidence for a marker's predictive or prognostic clinical utility under appropriate conditions, including careful pre-specification of the statistical analysis plan for evaluation of the marker [8].

A risky practice in the evaluation of predictive markers is to look for an association of a marker with clinical outcome by studying only patients who receive the new therapy. The problem with this approach is that prognostic effects can be confused with predictive effects. Suppose, for example, that the marker under study has a substantial prognostic effect so that patients with high levels of the marker will have better clinical outcome than patients with low levels of the marker regardless of what treatment the patient receives. Looking only at the patients treated with the new therapy might lead one to conclude erroneously that the improved outcome for patients with high levels of the marker was due to a preferential benefit of the new therapy for that marker-defined subgroup when, in fact, it is possible that the new therapy benefits no patients. Results of these types of studies can be misleading in the opposite direction as well. This can occur if the marker predicts for poor outcome under standard therapy, but patients with this marker benefit from the new therapy. The marker might exhibit no association with clinical outcome in the setting of new therapy, but only because the outcome for patients who were positive for the marker had been improved by the new therapy to be equivalent to the outcome for patients who were not positive for the marker. These examples underscore the need for examination of an appropriate control group (placebo or standard of care) when evaluating a potential predictive marker. A randomized trial provides an ideal setting to ensure that no other confounding factors influenced which patients received the standard therapy versus the new therapy.

### Extensive exploratory data analyses may result in spurious findings

Often extensive data analyses are performed in prognostic and predictive studies in a quest for associations between markers and clinical outcomes that demonstrate statistically significant *P *values. With the possibility to test association of multiple markers with multiple clinical endpoints in several patient subgroups, the chance of generating spuriously significant results in retrospective prognostic and predictive marker studies can be substantial [9].

Consider testing the association between a marker and a clinical outcome in each of four disjoint patient subgroups. If each statistical test is performed at the usual significance level of 0.05, the probability that a statistically significant result will be obtained in at least one of the four subgroups is 19%. Now consider multiple types of clinical outcomes and multiple markers and multiple cut-points applied to dichotomize continuous markers, and the likelihood of such a study producing at least one statistically significant result by chance can become very large. A similar problem occurs when treatment differences are tested in a clinical trial comparing two or more treatments arms in a multitude of subsets defined by markers or other patient characteristics. If treatment differences are found in some subsets and not others, investigators are tempted to claim that they have identified predictive subgroups. Most often such findings are spurious due to the multiple testing and are not confirmed in subsequent studies. Statisticians sometimes explain this phenomenon as 'if you torture the data long enough, they will confess to anything'. If these statistically significant findings are then retrofitted to a clinical question and published with no indication of the exploratory context in which the results were obtained, the result may represent a serious distortion of the significance (both statistical and clinical) of the findings. Together with the long recognized problem of publication bias favoring studies that report positive findings, the result may be a body of literature that is heavily influenced by false-positive findings.

### Sample sizes for adequate statistical power

Evaluation of predictive and prognostic markers using specimens collected within treatment trials is not a panacea, however. When designing clinical trials, sample size is generally determined to permit sufficient statistical power to detect a treatment effect of a pre-specified size. Often marker questions are either not specified during the planning stage for a therapeutic trial, or if they are, they are usually relegated to secondary aims that the study might not be sized to address with high statistical power. Add to that an inability to collect specimens from some patients in the trial, and statistical power can be diminished further. Major determinants of statistical power for analyses examining prognostic and predictive markers and their association with time-to-event endpoints (for example, time to disease recurrence or progression, or time to death) include the testing significance level (alpha or type I error), the expected number of events, the distribution of the marker (for example, positivity rate for a binary marker), the treatment randomization ratio, and the magnitude of effect.

Understanding the proper quantification of prognostic and predictive effects is important for determination of clinical utility and proper study design to evaluate those effects. For survival analyses, the effect of a binary prognostic marker is usually expressed as a hazard ratio. The relevant effect for a binary predictive marker is a treatment-by-marker interaction. Presence of a treatment-by-marker interaction means that the treatment effect, that is, the difference in clinical outcome between a new treatment and a standard treatment, differs depending on the status of the patient's marker. An interaction effect is often expressed in statistical terms as a ratio of the treatment hazard ratios, with one treatment hazard ratio being calculated in the marker 'positive' subgroup and the other treatment hazard ratio being calculated in the marker 'negative' subgroup. A treatment-by-marker interaction is most clinically relevant when it is a qualitative interaction. Qualitative means that the direction of treatment benefit is reversed in one marker subgroup compared to the other. For example, the new treatment might confer a substantial survival advantage to patients who are positive for the marker, but it may be the same or worse than standard treatment in the marker negative subgroup. Quantitative interactions occur when the treatment benefit is in the same direction but of different magnitude in the two patient subgroups. Unless the differential magnitude leads to a different treatment decision, a quantitative interaction may not translate to clinical utility of a test based on the marker.

The statistical power of a prognostic or predictive marker study depends on the distribution of the marker values in the patient population as well as the size of the effect that one aims to detect. When comparing survival between two groups of patients, for example, patients who receive two different treatments or patients whose tumors do versus do not express a particular marker, power is maximized when the groups are of equal size. Therefore, if a clinical trial has been designed with one-to-one randomization to detect a specified hazard ratio between treatment groups, a test for the effect of a binary prognostic marker on clinical outcome of the same magnitude as the treatment effect will have lower power than the treatment comparison when the binary marker has prevalence substantially different than 50%.

To test a marker-by-treatment interaction, the situation is even more challenging. To adequately power a clinical trial to test a treatment by marker interaction can easily require two to four times the sample size required to detect a treatment effect unless there is a fairly dramatic treatment effect nearly exclusive to the biomarker-predicted benefiting subgroup. Importantly, the biomarker-defined subgroup should not be defined *post hoc *by exploratory analyses and then tested as though it had been pre-specified unless proper care has been taken to statistically adjust for this form of multiple testing to avoid false-positive findings. An added problem is the inaccuracy of some marker assays. If assay inaccuracies cause misclassification of patients with regard to marker status, this error will cause further reduction in the statistical power for detecting predictive marker effects. Taken together, the considerations just discussed explain why it can be so difficult to establish utility of prognostic and predictive marker tests in a statistically rigorous way when the marker-related questions are retrofitted to therapeutics trials.

If there exist no suitable treatment trials with adequate specimen collection to adequately answer an important predictive or prognostic marker question, several options remain. These are to prospectively design a trial to specifically answer the marker question, or to try to combine specimens or marker data across several completed trials. Many options have been proposed for designing trials to validate marker-based tests [10], but such trials can be costly and currently are conducted less frequently than trials designed purely to answer a treatment question. Alternatively, combining over different marker studies might be possible, but care must be taken to select studies to represent the full spectrum of relevant studies, regardless of publication status or presence of statistically significant findings. Not only must patient characteristics and treatments be comparable in order for the studies to be combined sensibly, but the marker assays used in the different studies need to be comparable. All of these options require adequate resources, clear and unbiased reporting of studies, sharing of data, and potentially sharing of specimens.

## Conclusions

As marker-based clinical tests play an increasingly more prominent role in therapeutic decision-making, correspondingly greater attention needs to be paid to scientific rigor and clinical relevance earlier in the development and in the validation of such tests. This will require formation of collaborative teams including participation by laboratory scientists, technology developers, clinicians, statisticians, and bioinformaticians. Earlier emphasis should be placed on quality standards for specimen and data collection and pre-specified statistical analysis plans so that markers do not languish in never-ending cycles of confusing exploratory or poorly designed or analyzed studies. A comprehensive biomarker study registry has been proposed [11] and is currently under development. It is hoped that such a registry will make it easier to assess the body of evidence that already exists, and to identify studies that might be relevant for systematic reviews and meta-analyses. Studies need to be reported more thoroughly and transparently so that study quality and relevance can be judged more easily. Reporting guidelines for a variety of different types of biomedical research studies are now available http://www.equator-network.org/research-projects/. Specifically for tumor marker studies, the REMARK guidelines [12,13] provide useful guidance for reporting. The BRISQ guidelines [14] provide helpful guidance for reporting information about specimens used in biomedical studies. These efforts will provide a more representative body of evidence upon which to base decisions about the clinical utility of marker-based tests.

## Competing interests

The author declares that they have no competing interests.

## Authors' information

LM is a senior Mathematical Statistician in the Biometric Research Branch in the Division of Cancer Treatment and Diagnosis at the US National Cancer Institute where she works closely with the Cancer Diagnosis Program and Cancer Therapy Evaluation Program. Her statistical and collaborative interests and publications cover diverse topics including prognostic and predictive markers, genomic profiling, statistical design of prognostic and predictive marker studies, methods for the analysis of high-dimensional genomic data, multiple comparisons methods, surrogate endpoints, measurement error adjustment methods, and laboratory quality control and assay reproducibility assessment. She co-led the group that authored the REMARK guidelines for reporting tumor marker studies and is a co-author of the book *Design and Analysis of DNA Microarray Investigations*. Currently LM is a member of the Scientific Advisory Board of *Science Translational Medicine*.

## Pre-publication history

The pre-publication history for this paper can be accessed here:

http://www.biomedcentral.com/1741-7015/10/52/prepub

## References

[B1] Schilsky RS Personalizing cancer care: American Society of Clinical Oncology presidential address 2009 J Clin Oncol 2009 27 3725 3730 10.1200/JCO.2009.24.6827 19581526

[B2] Gardiner SJ Begg EJ Pharmacogenetics, drug-metabolizing enzymes, and clinical practice Pharmacol Rev 2006 58 521 590 10.1124/pr.58.3.6 16968950

[B3] Clark GM McShane LM Biostatistical considerations in development of biomarker-based tests to guide treatment decisions Statistics in Biopharmaceutical Research 2011 3 549 560 10.1198/sbr.2011.09038

[B4] Harris L Fritsche H Mennel R Norton L Ravdin P Taube S Somerfield MR Hayes DF Bast RC Jr American Society of Clinical Oncology 2007 update of recommendations for the use of tumor markers in breast cancer J Clin Oncol 2007 25 5287 5312 10.1200/JCO.2007.14.2364 17954709

[B5] Kyzas PA Denaxa-Kyza D Ioannidis JPA Almost all articles on cancer prognostic markers report statistically significant results Eur J Cancer 2007 43 2559 2579 10.1016/j.ejca.2007.08.030 17981458

[B6] Simon R Altman DG Statistical aspects of prognostic factor studies in oncology Br J Cancer 1994 69 979 985 10.1038/bjc.1994.192 8198989 PMC1969431

[B7] Simon R Development and validation of therapeutically relevant multi-gene biomarker classifiers J Natl Cancer Inst 2005 97 866 867 10.1093/jnci/dji168 15956642

[B8] Simon RM Paik S Hayes DF Use of archived specimens in evaluation of prognostic and predictive biomarkers J Natl Cancer Inst 2009 101 1446 1452 10.1093/jnci/djp335 19815849 PMC2782246

[B9] Ioannidis JPA Limits to forecasting in personalized medicine: An overview Int J Forecast 2009 25 773 783 10.1016/j.ijforecast.2009.05.003

[B10] Freidlin B McShane LM Korn EL Randomized clinical trials with biomarkers: design issues J Natl Cancer Inst 2010 102 152 160 10.1093/jnci/djp477 20075367 PMC2911042

[B11] Andre F McShane LM Michiels S Ransohoff DF Altman DG Reis-Filho JS Hayes DF Pusztai L Biomarker studies: a call for a comprehensive biomarker study registry Nat Rev Clin Oncol 2011 8 171 176 10.1038/nrclinonc.2011.4 21364690

[B12] McShane LM Altman DG Sauerbrei W Taube SE Gion M Clark GM Statistics Subcommittee of the NCIC-EORTC Working Group on Cancer Diagnostics REporting recommendations for tumor MARKer prognostic studies (REMARK) J Natl Cancer Inst 2005 97 1180 1184 10.1093/jnci/dji237 16106022

[B13] Altman DG McShane LM Sauerbrei W Taube SE Reporting recommendations for tumor marker prognostic studies (REMARK): explanation and elaboration BMC Medicine 2012 10 51 10.1186/1741-7015-10-51 22642691 PMC3362748

[B14] Moore HM Kelly AB Jewell SD McShane LM Clark DP Greenspan R Hayes DF Hainaut P Kim P Mansfield EA Potapova O Riegman P Rubinstein Y Seijo E Somiari S Watson P Weier H-U Zhu C Vaught J Biospecimen Reporting for Improved Study Quality (BRISQ) Cancer Cytopathol 2011 119 92 101 10.1002/cncy.20147 21433001

